# Evolutionary conservation of essential and highly expressed genes in *Pseudomonas aeruginosa*

**DOI:** 10.1186/1471-2164-11-234

**Published:** 2010-04-09

**Authors:** Andreas Dötsch, Frank Klawonn, Michael Jarek, Maren Scharfe, Helmut Blöcker, Susanne Häussler

**Affiliations:** 1 Chronic Pseudomonas Infections Research Group, Helmholtz-Center for Infection Research, Braunschweig, Germany; 2 Department of Genome Research, Helmholtz-Center for Infection Research, Braunschweig, Germany; 3 Project Group Bioinformatics and Statistics, Helmholtz-Center for Infection Research, Braunschweig, Germany; 4 Department of Computer Science, University of Applied Sciences Braunschweig/Wolfenbüttel, Wolfenbüttel, Germany; 5 Twincore, Center for Experimental and Clinical Infection Research, joint venture of the Helmholtz Center for Infection Research and the Medical School Hannover, Hannover, Germany

## Abstract

**Background:**

The constant increase in development and spread of bacterial resistance to antibiotics poses a serious threat to human health. New sequencing technologies are now on the horizon that will yield massive increases in our capacity for DNA sequencing and will revolutionize the drug discovery process. Since essential genes are promising novel antibiotic targets, the prediction of gene essentiality based on genomic information has become a major focus.

**Results:**

In this study we demonstrate that pooled sequencing is applicable for the analysis of sequence variations of strain collections with more than 10 individual isolates. Pooled sequencing of 36 clinical *Pseudomonas aeruginosa *isolates revealed that essential and highly expressed proteins evolve at lower rates, whereas extracellular proteins evolve at higher rates. We furthermore refined the list of experimentally essential *P. aeruginosa *genes, and identified 980 genes that show no sequence variation at all. Among the conserved nonessential genes we found several that are involved in regulation, motility and virulence, indicating that they represent factors of evolutionary importance for the lifestyle of a successful environmental bacterium and opportunistic pathogen.

**Conclusion:**

The detailed analysis of a comprehensive set of *P. aeruginosa *genomes in this study clearly disclosed detailed information of the genomic makeup and revealed a large set of highly conserved genes that play an important role for the lifestyle of this microorganism. Sequencing strain collections enables for a detailed and extensive identification of sequence variations as potential bacterial adaptation processes, e.g., during the development of antibiotic resistance in the clinical setting and thus may be the basis to uncover putative targets for novel treatment strategies.

## Background

In the face of the global emergence of multi-drug resistant bacterial pathogens, the search for new classes of antimicrobial agents is one of the most important challenges of modern medicine. Novel potential anti-bacterial drugs have mainly been discovered by conventional screening methods. These methods involved the testing of natural products or synthetic chemicals for growth inhibition or killing of wild-type test organisms, with the specific mode of action being worked out later [1-4]. However, recent advances in *in silico *genomic approaches have provided an opportunity to specifically highlight potential drug targets and have facilitated a paradigm shift from direct antimicrobial screening programs toward rational target-based strategies, where drug discovery starts at the level of the gene [4-7]. Fundamental improvements of genome-based technologies such as whole genome expression- and protein-profiling as well as whole genome sequencing has lead to further changes in the drug discovery process. This is due to the fact that large amounts of relevant biological information have become available to address highly complex biological questions [8-10].

As essential genes provide perfect potential drug targets, it has been claimed that an important task of rational target validation would be the identification of the essentiality of the genes within the genome of one organism [6]. There are several techniques to identify essential genes. First, experimental genetic inactivation of a potential target can be accomplished by gene disruption [11], either in a case-to-case approach [12] or in a high throughput mode [13,14] in order to provide a genome-wide assessment of essential genes in an organism. When interpreting genetic inactivation data it should, however, be recognized that the inability to isolate a viable stain under standard laboratory conditions is generally judged as evidence of essentiality, albeit these conditions might not reflect the growth conditions in, e.g., the host environment. In addition to the experimental validation of gene essentiality, information can be received by applying comparative genomics which involves the comparison of multiple fully sequenced genomes in order to identify a minimal genome set necessary to support bacterial viability [15,16]. This bioinformatic strategy assumes that bacteria accomplish essential functions through common mechanisms and that the genes encoding these functions would be highly conserved. An alternative approach for the prediction of gene essentiality would be to draw information from the sequence of the gene itself. The detailed bioinformatic analysis of the genomic makeup of one organism might give all the information necessary to classify a gene.

Although it was suggested that there is no difference between the rates of evolution for essential and non-essential genes in eukaryotes, Jordan et al. [17] demonstrated that essential genes appeared to be more conserved than non-essential genes, based on the analysis of multiple complete genomic sequences and experimental knock out data of three bacterial species. However, other studies have suggested an important direct influence of the expression levels on the rate of nonsynonymous substitutions in bacteria and demonstrated that when a control for this variable was included, essentiality played no significant role in the rate of protein evolution [18].

In this study we aimed to address the main issue of whether essential genes are more evolutionarily conserved than nonessential ones in bacteria and analyzed the impact of the gene expression rate and subcellular localization of the encoded proteins as additional factors that are linked to protein evolution. A remarkable collection of genomic data is already available for the opportunistic pathogen *Pseudomonas aeruginosa *[19]. Sequence information of six strains has been published [20-22,24], and there is a large collection of transcriptional profiles that have been recorded under various environmental conditions [25]. Furthermore, comprehensive experimental knock out libraries of two *P. aeruginosa *strains have been established [13,14].

In this study we amended the existing collection of *P. aeruginosa *genomic data with whole-genome sequence data from 36 clinical *P. aeruginosa *strains in order to accurately and definitively revisit the interdependence of gene essentiality, rate of nonsynonymous substitutions, gene expression and subcellular localization.

## Results and Discussion

### Pooled sequencing of 36 clinical *P. aeruginosa *isolates

In order to analyze the sequence variation of *P. aeruginosa *on a genome wide scale, a collection of 36 strains, merged to three 'sequence pools' (additional file 1), was sequenced on an Illumina Genome Analyzer. Additionally, four single strains (also included in one of the sequence pools) were sequenced individually, so that the accuracy of results obtained from the pooled samples could be analyzed. Pooling DNA from different strains - instead of sequencing all 36 genomes individually - was demonstrated to efficiently yield a large amount of data with an economically reasonable effort. Since we were mainly interested in the variation of the *P. aeruginosa *core genome [21], the obtained sequence fragments (35 bp Illumina paired end reads) were mapped to the genome of the reference strain PAO1 [23]. Genome coverage was sufficient for all sequencing runs with read depths (whole genome average) of 30.8 to 63.8 (Table 1), with the exception of some regions showing significantly decreased read depths. Such sequencing 'holes' indicate regions that are absent from the respective strain or - in case of sequence pools - from a fraction of the pooled strains. Short read data from all sequencing runs have been deposited at the NCBI sequence read archive (SRA) with the accession number 'SRP001802'.

**Table 1:** Overview of sequencing results.

	genome coverage		sequence variations	
Strain	median read depth^a^	missing genes^b^	nonsynonymous substitution *dN*^c^	synonymous substitution *dS*^c^
Psae1152	47.6	226	2.47	20.00
Psae1747	41.6	170	1.45	10.55
Psae2136	63.8	170	1.53	10.93
Psae2162	43.8	230	2.39	19.67

PADJ-1	36.7	61	3.26	11.40
PADJ-2	38.2	122	3.36	11.33
PADJ-3	30.8	93	3.13	11.07
mean^d^	34.6	87	3.22	11.25

^a ^Median value over all genes

^b ^Normalized read depth ≤ 0.25

^c ^Mean substitution rate per 1000 sites

^d ^Data set including all 36 strains obtained by merging short reads of the three PADJ sets.

### Sequence variation in *P. aeruginosa*

The median sequence variation (for protein coding genes in all 36 strains) was calculated to be ~0.47%, which is consistent with previous reports that found sequence variation in *P. aeruginosa *to be about one order of magnitude lower than in other γ-proteobacteria [26,27]. Nucleotide substitutions in genes coding for proteins can be either synonymous (do not change the amino acid sequence, also called silent substitutions), or non-synonymous (change the amino acid sequence). The median number of non-synonymous differences *dN *for all protein coding genes was 2.1 × 10^-3 ^with a ratio of nonsynonymous to synonymous (*dS*) substitutions of *dN*:*dS *= 0.19, indicating that variations of amino acid sequences are generally suppressed by selection.

It is well known that the *P. aeruginosa *core genome is highly conserved and there are only few exceptions of highly variable genes (e.g., *pilA *or the pyoverdin cluster, [26]), while inter-strain variation is mostly restricted to the accessory genome including pathogenicity islands and prophages [28]. Consequently, sequence variation among the 36 clinical *P. aeruginosa *strains was markedly increased (*dN *= 3.5 × 10^-3^) for genes belonging to these *regions of genomic plasticity *(RGPs, [21]).

### Essential genes in *P. aeruginosa*

According to the generally used definition, essential genes cannot be deleted from an organism without a lethal effect. Following this definition, the essential subset of genes within the *P. aeruginosa *genome has been determined [29] from a comparison of the two comprehensive transposon mutant libraries that are currently available for the two reference strains PAO1 [14] and PA14 [13]. For a total number of 335 genes, no transposon insertion was detected in either project [13]. However, three of these 335 putative essential genes were absent in at least two of the four individually sequenced genomes (Table 2): PA0637, which belongs to a cluster of genes (PA0616 - PA0648) encoding hypothetical phage-related proteins; PA2387 (*fpvI*), encoding a transcriptional regulator involved in pyoverdin mediated iron-uptake; and PA2565, encoding an unclassified hypothetical protein. Furthermore, we found that the normalized read depth was significantly reduced for these three genes in at least one of the sequence pools indicating that these three genes are not ubiquitously present in all strains and thus are most likely not essential for *P. aeruginosa*.

**Table 2:** Genes missing in the sequenced clinical isolates for which no transposon mutants are available^a^.

Locus ID	Psae1152/2162	Psae1747/2136	PADJ-1	PADJ-2	PADJ-3
normalized read depth^b^					
PA0637	0/0 (absent)	1.20/0.99 (present)	0.40	0.13	0.29
PA2387	0.18/0.18 (absent)	0.19/0.11 (absent)	0.26	0.89	0.47
PA2565	1.30/1.32 (present)	0/0 (absent)	0.79	0.89	0.47

^a ^Within the scope of the two comprehensive libraries [13,14].

^b ^Genes were assumed to be absent if the normalized read depth was below 0.25. In the pooled sequence data sets, the read depth gives only a rough estimate for the fraction of strains that harbor the gene.

### Codon adaptation index in *P. aeruginosa*

**Figure 1:**
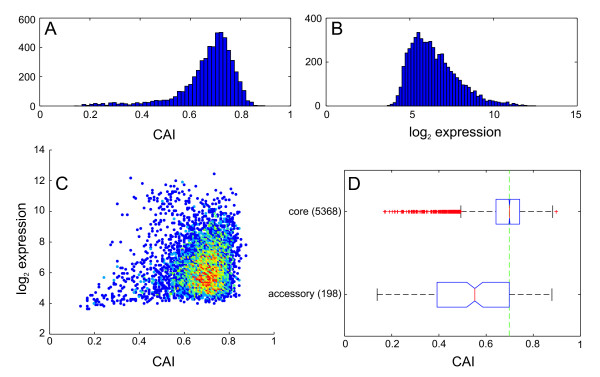
**Codon adaptation index is high in *P. aeruginosa *and independent of gene expression**. A) Histogram of codon adaptation index (CAI) and B) gene expression in *P. aeruginosa*. C) Gene expression data is only weakly correlated with the codon adaptation index in PAO1 (R = 0.135). Color indicates the density of data points at the coordinates indicated by the spots ranging from blue (single data point) to deep red (many). D) Core genes in PAO1 show generally high CAI values, while the accessory genome is significantly less adapted. Boxplots indicate 0.25 and 0.75 quantiles and the median (red line) with notches giving an estimate for the variance of the median. The green dashed line indicates the median *dN *for all genes.

In a previous attempt to link gene essentiality with the gene expression level, the codon adaptation index (CAI, [30]) was used as an approximation for gene expression [18] which is based on the assumption that highly expressed genes are generally optimized for translation speed and accuracy [31]. Essential genes were found to have a generally increased CAI as an indicator for their increased expression in *E. coli *and *B. subtilis *[18]. Here we used expression data of 232 microarrays from 27 independent experiments (downloaded from the NCBI Gene Expression Omnibus (GEO) website [25]) to get an estimate of the average gene expression level of each *P. aeruginosa *gene and compared gene expression and CAI for all genes of the *P. aeruginosa *genome. The bias caused by the specific setup of individual transcriptome experiments was assumed to be minimized by the large number of independent data sets that where included in this calculation. Indeed, the CAI is generally very high (Figure 1A), which is not reflected in a global increase in gene expression (Figure 1B). Additionally, correlation of CAI and gene expression was found to be weak (Figure 1C), supporting the previous findings of Kiewitz and Tümmler [27] who showed that the codon usage is globally optimized in *P. aeruginosa *and not restricted to only highly expressed genes pointing towards a generally high level of adaptation throughout the genome that would ensure optimal gene expression under various environmental and metabolic conditions. In consistence, we found the CAI to be significantly reduced in genes of the accessory genome (Figure 1D), which in many cases were acquired horizontally and also differ from the core genome in several other aspects (e.g., GC content).

### Gene essentiality and expression level are correlated with genetic variability

**Figure 2:**
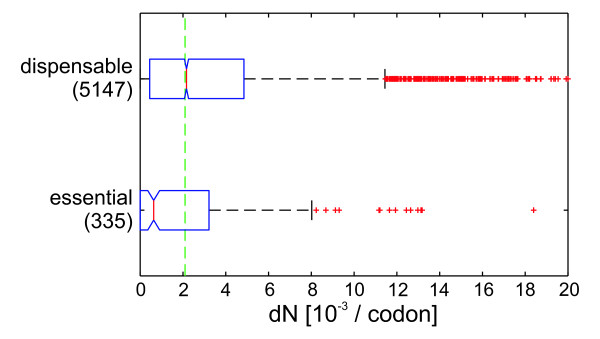
**Protein evolution in essential and dispensable genes**. Boxplots of nonsynonymous substitution rates *dN *of coding sequences averaged for all 36 strains. Data were grouped by gene essentiality as annotated in the Database of Essential Genes [13]. 87 genes showing a very low read depth (≤ 25% of the average read depth) were neglected in this analysis. Boxplots indicate 0.25 and 0.75 quantiles and the median (red line) with notches giving an estimate for the variance of the median. The green dashed line indicates the median *dN *for all genes. Values with *dN *above 20 × 10^-3 ^are not shown.

**Figure 3:**
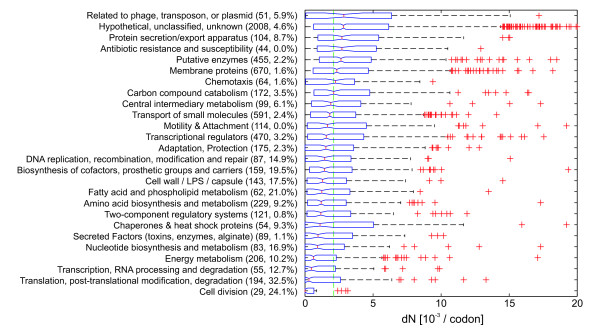
**Protein evolution rates in genes of different functional categories**. Boxplots of nonsynonymous substitution rates *dN *of coding sequences averaged for all 36 strains. Data were grouped by functional category according to PseudoCAP functional categories [19]. Numbers in brackets indicate the number of genes and the percentage of essential genes for each functional category. Groups were sorted by descending median *dN*. 87 genes showing a very low read depth (≤ 25% of the average read depth) were neglected in this analysis. Boxplots indicate 0.25 and 0.75 quantiles and the median (red line) with notches giving an estimate for the variance of the median. The green dashed line indicates the median *dN *for all genes. Values with *dN *above 20 × 10^-3 ^are not shown.

It has previously been shown that essential genes are more evolutionarily conserved than nonessential genes [17], and indeed determination of non-synonymous and synonymous substitution rates *dN *and *dS *for the 36 clinical *P. aeruginosa *strains confirmed this expected correlation of sequence variation and essentiality (Figure 2). If genes are grouped by their functional classification, the variation is lowest for genes involved in 'transcription, RNA processing, degradation', 'translation, post-translational modification, degradation' and 'cell division', which are enriched for essential genes (Figure 3).

**Figure 4:**
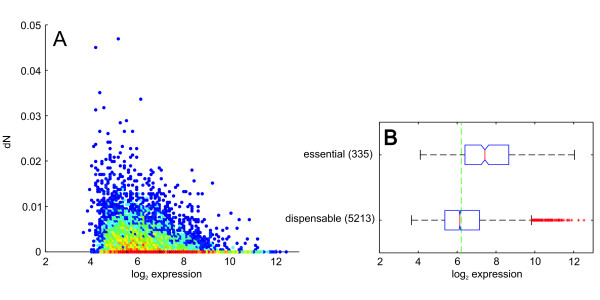
**Gene expression correlates with substitution rate and gene essentiality**. A) Nonsynonymous substitution rate *dN *(coding sequences averaged for all 36 strains) versus the expression level. Color indicates the density of data points ranging from blue (single data point) to deep red. B) Boxplots of gene expression levels grouped by gene essentiality as annotated in the Database of Essential Genes [29]. Boxplots indicate 0.25 and 0.75 quantiles and the median (red line) with notches giving an estimate for the variance of the median. The green dashed line indicates the median *dN *for all genes.

In addition to gene essentiality, a high gene expression rate has previously been shown to correlate with low sequence variation [18], and it was proposed that the underlying driving force for the slower evolution of essential genes is that most of the highly expressed genes are also generally indispensable. To test this for *P. aeruginosa*, we compared the gene expression data of *P. aeruginosa *(averaged from 232 microarrays as described above) with the rate of nonsynonymous substitutions, *dN *(Figure 4A). Applying robust regression to predict *dN *from gene expression clearly demonstrated a negative correlation (not shown), which means that highly expressed genes are indeed more conserved in *P. aeruginosa*.

Furthermore, essential genes showed a significantly increased expression level in *P. aeruginosa*. If the gene expression data are grouped by essentiality it becomes clear, that essential genes show a significant increased proportion of highly expressed genes (Figure 4B). With the aim of testing whether the observed correlation of gene essentiality and *dN *is the result of this overrepresentation of high expression rates among the essential genes or whether there is also an independent effect, we performed a statistical test for conditional independence. Therefore, the *dN*-values of the non-essential genes were normalized in order to compensate for their lower expression rates. The null hypothesis for the test for conditional independence was that the distributions of the *dN*-values are identical for essential and non-essential genes after this normalization has been performed. The null hypothesis of conditional independence had to be rejected for the averaged data set of all 36 strains (p = 0.0169). This means that, besides the expression level, essentiality also accounts for *dN *in *P. aeruginosa*, although the statistical significance is not very high. Apparently, the effect of gene expression on protein evolution rates is dominant in *P. aeruginosa*, but still an independent (though weaker) effect of essentiality could be observed.

### Extracellular proteins evolve at a faster rate

Other parameters that have been identified to correlate with slow protein evolution include protein localization (with extracellular proteins being more variable than cytoplasmic ones) [32-34], evolutionary age [34] and protein connectivity [33].

**Figure 5:**
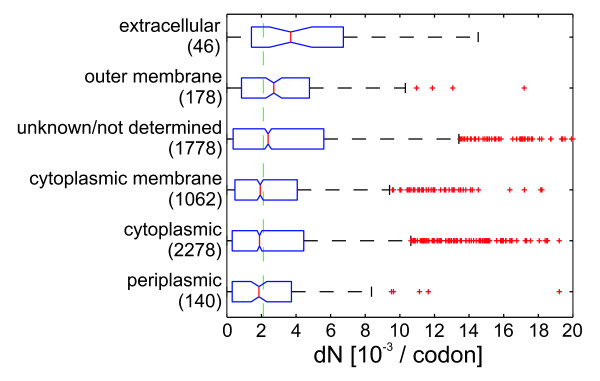
**Protein evolution in genes with different subcellular localization**. Boxplots of nonsynonymous substitution rates *dN *of coding sequences averaged for all 36 strains. Data were grouped by subcellular localization as annotated in the *Pseudomonas *genome database [19]. 87 genes showing a very low read depth (≤ 25% of the average read depth) were neglected in this analysis. Boxplots indicate 0.25 and 0.75 quantiles and the median (red line) with notches giving an estimate for the variance of the median. The green dashed line indicates the median *dN *for all genes. Values with *dN *above 20 × 10^-3 ^are not shown.

In *P. aeruginosa *genes encoding extracellular and outer membrane proteins ('extracellular genes') showed a significant increase in *dN *as compared to cytoplasmic genes (Figure 5). We also repeated robust regression for the *dN *and expression data for each class of subcellular localization independently. The aforementioned negative correlation of *dN *and gene expression could be found for all localization classes with the exception of extracellular genes. On the contrary, extracellular genes even had a slightly positive coefficient indicating that they are even more variable if they are highly expressed (not shown).

### Highly conserved non-essential genes

As described above and depicted in Figure 2, essential genes were shown to be generally more conserved than non-essential ones in our collection of *P. aeruginosa *strains. Vice versa, many highly conserved genes were essential according to the standard definition (*experimentally essential*) because no transposon mutants are available in either of the two comprehensive libraries [13,14]. However, we also found a large subset of genes that are experimentally dispensable but highly conserved. Genes that are dispensable, but conserved throughout distantly related bacteria, have been termed persistent non-essential genes and are proposed to be regarded as truly essential from an evolutionary point of view [35]. These genes might be dispensable for short term survival and growth under laboratory conditions but are in fact essential for successful survival of the population under varying environmental conditions in its native habitat.

Interestingly, overall as many as 980 protein encoding genes (only 124 of which were experimentally essential) did not exhibit any variations in the protein sequence (*dN *= 0, *completely conserved genes*) in the 36 clinical isolates and 79 were even identical at the nucleotide level (*dS *= 0). While this is consistent with the generally high conservation of the *P. aeruginosa *core genome, these genes could furthermore be interpreted as being 'evolutionarily essential' for the species *P. aeruginosa *in a sense similar to the aforementioned persistent non-essential genes [35].

Among the 980 completely conserved genes (additional file 2) we found many encoding ribosomal proteins, genes important for energy production (respiratory chain, ATP synthase) or DNA replication and repair, e.g., *dnaN *(encoding for a DNA polymerase III subunit) or the helicase *ruvA*/*ruvB*. Many of them also belong to the class of persistent non-essential genes in γ-Proteobacteria [35].

Remarkably, among the set of completely conserved genes we also found the two paralogous operons *phzA1-G1 *and *phzA2-G2 *that encode enzymes necessary for phenazine synthesis [36]. Both operons are almost identical, not only within one strain (with only a few dissimilarities between genes *phzA1/2*, *phzB1/2 *and *phzC1/2 *in PAO1) but furthermore no sequence variation could be detected for the whole collection of 36 clinical strains (with the only exceptions of *phzA1 *and *phzB2*) - in most cases not even at the nucleotide level (*dS *= 0). Phenazines including pyocyanin, which is responsible for the well known blue-green color of *P. aeruginosa *cultures, and which is a major virulence factor [37], have been shown to mediate extracellular electron transfer under microaerophilic conditions [38]. Other genes involved in phenazine synthesis and regulation (*phzM*, *mexG*, *pqsE*) and many regulatory genes - *vfr*, *algU*, *rsmA*, *gacA*, *phoP*, *mvaT*, *algR*, and *phoB*, all of which play an important role in the environmental versatility of *P. aeruginosa *including virulence [39,40] - also showed no protein variations.

Additionally, we identified the quorum sensing (QS) genes *lasI*, *rhlR *and *rsaL *to be fully conserved at the protein level. It has been reported that QS is frequently impaired in clinical isolates, especially those isolated from long term chronically infected cystic fibrosis (CF) patients [41-43]. Thereby, loss of QS is mainly caused by disruption of *lasR *that also showed considerable variation in this study, including the identification of premature *stop*-codons in a fraction of strains (not shown). While the high conservation of the above described genes of the *las *and *rhl *system underlines the general importance of QS for *P. aeruginosa*, loss of QS - preferably by disruption of *lasR *- might be an important adaptive strategy to more specific habitats such as the chronically infected CF lung.

### Highly conserved genes linked to antibiotic resistance

Assuming that high conservation of genes is positively correlated with their evolutionary importance, the 980 highly conserved genes described above might be potential novel drug targets. To further examine this potential, we compared the list of genes with the results of three recently published screens for resistance determinants in *P. aeruginosa *[44-46]. In total, of the 980 genes 27 were identified in at least one of the three screens as being positively linked to antibiotic resistance (additional file 2).

Since non-specific drugs can cause more side-effects, potential new drug targets should be specific for the bacteria targeted. Thus, in order to determine the specificity of the highly conserved genes with respect to their phylogenetic distribution, we performed a BLASTP search of all 980 protein sequences against the database of non-redundant protein sequences (nr) obtained from the NCBI BLAST website http://blast.ncbi.nlm.nih.gov/. 56 genes were found to be specific to *Pseudomonas*, this included 24 species-specific genes for which no orthologs outside *P. aeruginosa *were identified (Table 3). For the majority of genes a large number of orthologs could be identified that belonged to a variety of species outside the *Pseudomonas *genus indicating a broad phylogenetic distribution among different bacterial genera. At least one human ortholog was identified for 6 of the 980 genes (additional file 2), which might be adversely affected by a drug.

**Table 3:** Genes completely conserved on the protein level that are specific to the genus *Pseudomonas*

Locus tag	Gene name	Locus tag	Gene name	Locus tag	Gene name
PA0033^b^		PA2381^b^		PA4035^a^	
PA0050^a^		PA2447^a^		PA4139^b^	
PA0052^b^		PA2486^b^		PA4303^a^	*tadZ*
PA0061^a^		PA2747^b^		PA4360^b^	
PA0377^b^		PA2766^b^		PA4556^a^	*pilE*
PA0776^b^		PA2781^b^		PA4570^a^	
PA1000^b^	*pqsE*	PA2808^b^	*ptrA*	PA4608^a^	
PA1021^b^		PA2884^b^		PA4643^a^	
PA1064^a^		PA2894^b^		PA4685^a^	
PA1103^a^	*fliH*	PA2896^a^		PA4697^a^	
PA1347^b^		PA2897^a^		PA4815^a^	
PA1414^b^		PA2899^b^		PA4843^a^	
PA1442^a^	*fliL*	PA3062^a^	*pelC*	PA5052^a^	
PA1531^a^		PA3280^a^	*oprO*	PA5295^a^	
PA1621^a^		PA3309^b^	*uspK*	PA5364^a^	
PA1753^a^		PA3332^b^		PA5424^b^	*yeaQ*
PA1773^a^	*cmaX*	PA3423^a^			
PA1921^b^		PA3486^a^			
PA1970^a^		PA3515^b^			
PA2332^a^		PA4032^a^			

^a ^no orthologous genes were found outside the genus *Pseudomonas*.

^b ^no orthologous genes were found outside the species *P. aeruginosa*

## Conclusions

Thanks to recent advances in sequencing technologies it has been possible to analyze many genomes with little expense within a short space of time. Because whole-genome sequence data and bioinformatics provide potential for large scale comparative genomics and evolutionary inference, they will be an important sector in the future global approaches for deciphering the genomic make-up of an organism and for rational drug discovery programs. In the presented work, we have demonstrated the combined use of short read sequencing and bioinformatic methods to analyze a whole collection of clinical *P. aeruginosa *strains. The genome sequence data of 36 pooled bacterial strains combined with the availability of more than 200 gene expression profiles and the experimental genome-wide assessment of essential genes, gave the unique opportunity to revisit the central question of the interdependency of the effects of various factors on sequence variation in an accurate and definite manner. Here, we have confirmed that the rate of expression is a major determinant of how rapidly a protein evolves. We were furthermore able to demonstrate an independent correlation of gene essentiality with protein evolution rates in *P. aeruginosa*, although essential genes on average also showed an increased expression. Additionally, we could demonstrate increased substitution rates for genes encoding for extracellular proteins.

Furthermore, our comparison of whole genome sequence data of 36 *P. aeruginosa *strains revealed a subset of 980 proteins that were fully conserved, and did not show any variation in the amino acid sequence. The full conservation of these protein sequences may indicate their evolutionary importance for *P. aeruginosa *as a species, comparable to the persistent nonessential genes on the inter-species level. Among the completely conserved proteins, we found many that are involved in the central cellular mechanisms like energy production, replication and protein synthesis, many of which were also identified by Fang et al. as persistent nonessential genes, or that are coding for regulatory and virulence factors (additional file 2). In accordance with the lifestyle of *P. aeruginosa *as a versatile environmental organism and opportunistic pathogen, this demonstrates a) the high level of optimization of the genome (which is also obvious in the codon bias, Figure 1) and b) the evolutionary importance of these genes for its survival in various habitats.

The genomic analysis of multiple strains will greatly enhance our knowledge on the distribution and variation of genes and their relation to the lifestyle of the particular organism. A future expansion of this study, which is biased towards clinical isolates, would be to include soil and water isolated *P. aeruginosa *strains and to test whether they share the identified traits. Furthermore, the comparison of strain collections with specific virulence or resistance phenotypes will simplify the detection of genetic determinants that are responsible for the development of severe infections and/or multi-resistance and will uncover putative targets for novel anti-bacterial strategies.

## Methods

### Organism & Culturing

A collection of 36 clinical *P. aeruginosa *isolates (additional file 1) was used for sequencing. The strains were divided into 3 groups and sequenced as pooled samples. Group 1 contained fourteen strains, group 2 seven strains and group 3 fifteen strains. Out of group 1, four strains (Psae1152, Psae1747, Psae2136 and Psae2162) were selected to be also sequenced individually. Each strain was cultivated independently in 5 mL LB medium in 50 mL glass flasks at 37°C, 180 rpm over night. Cells were harvested by centrifugation of 1 mL culture (5 min, 8000 rpm) in a tabletop microcentrifuge and washed once by resuspension in 1 mL H_2_O and repeated centrifugation.

For sequencing of pooled samples, all 36 isolates were grown independently in 2 mL LB medium in glass tubes at 37°C, 180 rpm over night. Cells were harvested by centrifugation of 1 mL culture (5 min, 8000 rpm), the supernatant and visible extracellular matrix material were removed and the remaining pellets were resuspended in 1 mL H_2_O. Cell suspensions were pooled according to their allocation to one of the three groups at equal cell numbers as estimated by determination of the optical density at 600 nm using a NanoDrop photometric device (ThermoScientific). The resulting pooled suspensions were centrifuged (5 min, 8000 rpm), supernatant was removed and the pellets were stored at -70°C.

### DNA preparation and sequencing

Genomic DNA was isolated from thawed pellets using the DNeasy Blood & Tissue Kit (Qiagen) according to the manufacturer's instructions. DNA samples were further prepared and sequenced using 35 bp paired end sequencing on a Genome Analyzer II (Illumina). Libraries of 300 bp prepared according the manufacturer's instructions "Preparing Samples for Paired-End-Sequencing". Cluster generation was performed using the Illumina cluster station, sequencing for read 1 and read 2 on the Genome Analyzer followed a standard protocol. The fluorescent images were processed to sequences using the Genome Analyzer Pipeline Analysis software 1.3.2 (Illumina).

The primary sequencing results (short read data) of the four individually sequenced strains and the three pools have been deposited in the NCBI Sequence Read Archive (SRA, http://www.ncbi.nlm.nih.gov/Traces/sra/sra.cgi) as a single study with the accession number 'SRP001802'.

### Sequence analysis

The sequence output (35 base pair short reads) of the Genome Analyzer II was transformed to FastQ format and mapped against the genome sequence of reference strain *P. aeruginosa *PAO1 downloaded from the Pseudomonas genome database [19] using the 'easyrun' option of the Maq software [47]. For the four single strains, genetic variations were identified from the 'consensus.snp' output file produced by Maq. SNPs were filtered by consensus quality (≥ 30) and read depth (≥ 15) to exclude low quality reads. For the pooled sequences, genetic variations were identified using a Perl script for SNP detection from pooled DNA samples that uses the -N option of Maq to calculate frequencies with confidence intervals for all SNPs [48]. To optimize parameter settings for *P. aeruginosa *sequence data, the results of the four individual samples were pooled *in silico *and analyzed using the aforementioned script with varying parameter sets (minimum mapping quality: 20 - 40, maximum mismatches per read: 1 - 5). The results were compared with the SNPs detected in the four individually sequenced strains. Using ≥ 25 mapping quality and ≤ 3 mismatches yielded a sensitivity of 89.8% and specificity (fraction of true positives) of 99.0%, therefore these parameters were applied as standard settings for SNP identification in the pooled sequences data.

In case of the four individually sequenced strains, the rates of synonymous (*dS*) and nonsynonymous (*dN*) substitution (relative to the PAO1 reference genome) were computed for all gene sequences based on the Goldman & Yang model [49] using MBEtoolbox for Matlab [50]. For the pooled sequencing data, these algorithms were not applicable because SNPs are not generally present in all strains. Therefore the substitution rates *dS *and *dN *were approximated by calculating the weighted average amount of substitutions per site (i.e., per codon):

where *dN*_*j*, *k *_and *dS*_*j*, *k *_are the nonsynonymous and synonymous substitution rate (per site/codon) for gene *j *in data set *k*, *l*_*j *_is the length of gene *j *(base pairs), *w*_*i *_is the weight (relative frequency) of SNP *i *and *a*_*i *_is 1 if the SNP constitutes a nonsynonymous substitution and 0 otherwise ('silent' mutations). To calculate the average values for the complete set of 36 strains, the values of each pooled sequence were weighted by the number of strains:

where  and  are the nonsynonymous and synonymous substitution rate averaged for all 36 strains, *s*_*k *_is the number of strains included in data set *k *and *dN*_*j*, *k *_and *dS*_*j*, *k *_are the respective rate for data set *k*.

Information on gene essentiality in *P. aeruginosa *was taken from the Database of Essential Genes [29]http://tubic.tju.edu.cn/deg/, which integrates the results of two comprehensive *P. aeruginosa *transposon mutant libraries [13,14]. Gene annotation data (PseudoCAP functional category, subcellular localization, etc.) were downloaded from the *Pseudomonas *genome database [19]http://www.pseudomonas.com/. Gene expression data were obtained from the NCBI Gene Expression Omnibus database [25]. The median expression for each gene was calculated from normalized absolute log_2 _signals of 232 microarrays (27 independent experiments) found in the database for the GPL84 platform (Affymetrix *P. aeruginosa *array Pae_G1a).

### Robust regression

Standard least squares regression is extremely sensitive to single outliers. Robust regression replaces the squared error by other error measures reducing the influence of outliers [51]. In order to avoid artifacts introduced by outliers with respect to dN or the expression level, we have preferred robust regression over standard least squares regression. Here we have applied robust regression based on Huber's error function as it is implemented in the statistics software R in the rlm-method in the package MASS.

### Statistical test for conditional independence

The random variable X is said to be conditionally independent of the random variable Y given the random variable Z if P(X|Y,Z) = P(X|Z) holds. Conditional independence means that X might be highly correlated with Y as well as Z, but the correlation between X and Y is fully explained or covered by Z. Here, we apply a Kolmogorov-Smirnov test to the distributions of the *dN*-values (corresponding to the random variable X) of the essential genes and the non-essential genes (essentiality/non-essentiality represents the random variable Y) where the *dN*-values of the non-essential genes are weighted according to the different expression levels (random variable Z) in essential and non-essential genes [52].

### Identification of orthologous proteins

*P. aeruginosa *PAO1 protein sequences obtained from the *Pseudomonas *Genome Database [19] were aligned to the 'nr' database of non-redundant protein sequences obtained from the NCBI blast website [53] using BLASTP. All the resulting alignments with at least 50% identities and an alignment length of at least 50% of the size of the PAO1 protein were considered as orthologs.

## Authors' contributions

AD, MJ, MS and HB performed the experiments and analyzed the data. FK supervised mathematical analysis of the sequencing results. SH designed the study. AD and SH wrote the manuscript.

## Supplementary Material

Additional file 1 **Overview of the clinical strains included in the sequenced pools**. This table specifies the strains that were merged in the three sequence pools and their clinical source. Click here for file

Additional file 2 **Complete list of proteins with full conservation of the amino acid sequence**. All 980 genes identified to show no sequence variation at the protein level (nonsynonymous substitution rate *dN *= 0) in all three sequence pools are listed in this xls file. Additional information includes gene annotation, experimental essentiality, phylogenetic distribution of orthologs and effect on antibiotic susceptibility. Click here for file
